# Editorial to “Initial experiences and technical insights of pulmonary vein isolation with FARAPULSE pulsed field ablation in patients implanted with WATCHMAN left atrial appendage closure devices: The first report in Japan”

**DOI:** 10.1002/joa3.70105

**Published:** 2025-06-16

**Authors:** Masato Fukunaga

**Affiliations:** ^1^ Department of Cardiology Kokura Memorial Hospital Kitakyushu Japan

**Keywords:** atrial fibrillation, left atrial appendage closure, pulsed field ablation

Editorial comment on “Initial experiences and technical insights of pulmonary vein isolation with FARAPULSE pulsed field ablation in patients implanted with WATCHMAN left atrial appendage closure devices: The first report in Japan.”^1^

Pulsed field ablation (PFA) has recently emerged as a promising technique for atrial fibrillation (AF) ablation, rapidly gaining popularity due to its favorable safety and efficacy profile. Unlike conventional thermal ablation, PFA devices exhibit significant variation in catheter design, workflow, and clinical evidence—differences that may be even more pronounced across platforms. Notably, among current systems, only the FARAPULSE PFA system (Boston Scientific) can be used in patients with a preexisting left atrial appendage closure (LAAC) device, according to the instructions for use. While PFA can theoretically be applied in such settings, metallic interference between the PFA catheter and LAAC device remains a potential procedural challenge.

In a recent issue of the *Journal of Arrhythmia*, Chatani et al.^1^ presented a small case series demonstrating the feasibility of PFA in patients with prior WATCHMAN device implantation (3 months–2 years postimplantation), under intracardiac echocardiography (ICE) guidance. In Case 2 of their report, catheter artifact interference was observed when the FARAWAVE catheter was configured in its flower formation. This issue was resolved by adjusting catheter depth and position posteriorly. The LAAC device in that case was implanted proximally, protruding 4.4 mm from the ridge of the left superior pulmonary vein. The authors also noted that the Amplatzer Amulet device may pose a higher risk of interference due to its design. Importantly, all cases were completed without procedural complications, supporting the feasibility of PFA in this population.

Despite this success, certain concerns persist. Metal artifact interference remains a technical hurdle, particularly in anatomically complex or combined procedures. The concept of a combined AF ablation and LAAC—often termed the “one‐stop procedure”—has gathered increasing interest. The OPTION trial evaluated LAAC as an alternative to oral anticoagulation in patients post‐AF ablation. The trial permitted both concomitant LAAC (within 10 days of ablation) and delayed LAAC (90–180 days postablation). Results demonstrated that LAAC was associated with a lower incidence of nonprocedure‐related major or clinically relevant nonmajor bleeding, and it was noninferior to oral anticoagulation for a composite endpoint of all‐cause death, stroke, or systemic embolism at 36 months.^2^ While these findings have not yet led to widespread procedural changes, the combined approach remains appealing to both clinicians and patients.

The ongoing OPTION‐A trial (NCT06686485), a prospective, single‐arm, multicenter postmarket study, is the first to formally evaluate the FARAPULSE PFA system and the WATCHMAN device in a concomitant setting. This study may provide critical insight into the viability of the “one‐stop procedure.” Still, several practical issues must be addressed before this approach becomes routine. First, ICE‐guided procedures, while feasible, can be technically demanding—especially with current 2D‐ICE technology. Although recent data suggest acceptable outcomes with ICE guidance, moderate peri‐device leak (PDL < 3 mm) rates remain relatively high.^3^ Second, according to multicenter registry data from Hong Kong, significant PDL (>3 mm) was more common in combined PFA‐LAAC procedures than in LAAC alone.^4^ This finding is hypothesized to result from the resolution of postablation pulmonary ridge edema, which may initially mask inadequate device sealing.

Taken together, current evidence raises several key clinical questions: (1) Should LAAC be performed concomitantly or sequentially following AF ablation? (2) Which patient populations are best suited for the one‐stop approach? (3) Is PFA truly the optimal energy source for combined procedures? (4) How can we optimize imaging and procedural workflow to minimize complications such as PDL? Although the ultimate goal appears within reach, additional evidence is essential to define the optimal clinical strategy.

## CONFLICT OF INTEREST STATEMENT

A proctor for Boston Scientific Japan, honorarium from Boston Scientific Japan.

## ETHICS STATEMENT

N/A.

## PERMISSION TO REPRODUCE MATERIAL FROM OTHER SOURCES

None.

## PATIENT CONSENT STATEMENT

N/A.

## CLINICAL TRIAL REGISTRATION

N/A.

## Data Availability

None.
